# Population Structure Based on Microsatellite Length Polymorphism, Antifungal Susceptibility Profile, and Enzymatic Activity of *Candida auris* Clinical Isolates in Russia

**DOI:** 10.3390/jof11010035

**Published:** 2025-01-04

**Authors:** Ellina Oganesyan, Victoria Klimenteva, Irina Vybornova, Valentina Venchakova, Ekaterina Parshikova, Sergey Kovyrshin, Olga Orlova, Alexander Kruglov, Svetlana Gordeeva, Natalya Vasilyeva, Anastasiya Taraskina

**Affiliations:** 1 Kashkin Research Institute of Medical Mycology, North-Western State Medical University Named after I.I. Mechnikov, 191015 Saint Petersburg, Russia; nabokavika97@mail.ru (V.K.); irina_vybornova@mail.ru (I.V.); sergei.kovyrshin@szgmu.ru (S.K.); natalya.vasileva@szgmu.ru (N.V.); ataraskina@mail.ru (A.T.); 2 Department of Medical Microbiology, North-Western State Medical University Named after I.I. Mechnikov, 191015 Saint Petersburg, Russia; valya.venchakova.98@mail.ru (V.V.); tereshkova-kata@mail.ru (E.P.); 3 Moscow L.A. Vorokhobov Municipal Clinical Hospital № 67, 123423 Moscow, Russia; o.e.orlova@yandex.ru; 4 Moscow Municipal Clinical Hospital № 40, 129301 Moscow, Russia; kruglov_a_n@mail.ru; 5 Clinical Infectious Diseases Hospital Named after S.P. Botkin, 195067 Saint Petersburg, Russia; svetalgor@mail.ru

**Keywords:** *Candida auris*, nosocomial outbreak, molecular epidemiology, microsatellite length polymorphism, antifungal susceptibility, phospholipase activity, esterase activity, epidemiological typing

## Abstract

*Candida auris* is an emerging multidrug-resistant fungal pathogen causing nosocomial transmission and invasive infections with high mortality. This study aimed to investigate the genetic relationships, enzymatic activities, and drug-resistance profiles of *C. auris* isolates to evaluate the population and epidemiological diversity of candidiasis in Russia. A total of 112 clinical isolates of *C. auris* were analyzed from May 2017 to March 2023 in 18 hospitals across Saint Petersburg, the Leningrad Region, and Moscow. Species identification was confirmed by ITS sequencing, and genotyping was performed using 12 short tandem repeat (STR) markers. Antifungal susceptibility was tested using Sensititre™ YeastOne™ plates, and hydrolytic enzyme production was measured by the plate method. ITS sequencing confirmed that all isolates belonged to a single ITS cluster (clades I and III). Fifteen distinct STR genotypes were identified, with genotype I being dominant (*n* = 53). The most variable of the analyzed markers turned out to be M3-Ia, which was represented in the Russian population by eight different variants. Fluconazole resistance was found in 111 isolates, 17% were resistant to amphotericin B, and 3.6% to 5-flucytosine. Phospholipase activity was strong in most strains, especially in urine isolates (*p* = 0.014). Conclusion: The predominance of STR genotype I and its variability at the M3-Ia locus suggest its association with nosocomial outbreaks and transmissibility in Russia.

## 1. Introduction

Invasive fungal infections due to opportunistic pathogens have become a major clinical and pharmaceutical challenge since the end of the 20th century, especially for the population of immunocompromised patients [1,2,3]. The introduction of new medical technologies into practice (surgical procedures, transplantation of organs and tissues, high-dosage cytostatic and immunosuppressive therapy, etc.), along with the HIV/AIDS pandemic, led to an increase in the frequency of systemic mycoses [1]. In the context of nosocomial invasive fungal infections, *Candida* species are the predominant causative agents, with mortality rates exceeding 40% [2,3].

Among fungal yeast infections, emerging species are a special threat, as they are often resistant to various antifungal drugs and present diagnostic challenges. The Centers for Disease Control and Prevention (CDC) and the World Health Organization (WHO) recently identified *Candida auris* as a high-priority health threat based on several key variables such as global mortality rates, availability of diagnostics, and reports of antifungal drug resistance [4,5].

*C. auris* belongs to the Metschnikowiaceae family, which includes species that are often resistant to multiple antifungal drug classes [6,7]. Molecular clock evaluation (based on spontaneous and evolutionary mutation rates) has demonstrated that the most recent common ancestor (TMRCA) of *C. auris* is older than 360 years, with clade II being the oldest. Estimates indicate that nearly all outbreak-causing clusters from clades I, III, and IV originated between 1982 and 1984, approximately 38 to 40 years ago. The occurrence of these clusters coincides with the introduction of azole drugs into clinical practice and the early stages of the AIDS epidemic [8,9]. However, the environmental reservoirs of this pathogen remain unknown. The salt marsh wetlands and sandy beaches of the Andaman Islands have been identified as natural niches for *C. auris* [10]. Additionally, yeast pathogens, including *C. auris*, have been found in dairy production facilities [11], though the connection between these findings and human infection remains unclear. It has been shown that the use of fungicides in the agricultural industry, particularly in fruit storage, is a significant factor driving the selection of drug resistance in clinical settings [12,13].

Currently, *C. auris* represents the most significant clinical challenge in controlling nosocomial infections globally. In addition to its increased multidrug resistance, this pathogen exhibits high transmissibility within healthcare facilities and can be transmitted from patient to patient, causing clusters of cases or outbreaks that are difficult to control, as this species persists in hospital environments, where it is difficult to eradicate [14,15]. Besides antifungal drug resistance, fungal virulence factors can also negatively impact disease outcomes and enhance the pathogen’s transmissibility. Proteolytic enzymes such as proteinase, aspartyl protease, hemolysins, lipases, and phospholipase are crucial for *C. auris* to adhere to the tissue surfaces and invade host cells [16]. Phospholipase and esterase enzymes play crucial roles in the virulence of *Candida* species, including *C. auris*. These enzymes facilitate the invasion of host tissues by degrading phospholipids and ester bonds in cellular membranes, thus enhancing pathogenicity [17]. Specifically, phospholipase activity has been associated with increased tissue adherence and invasion, while esterase activity aids in the breakdown of lipids essential for cellular membrane integrity. The examination of these enzymatic activities is therefore critical for understanding the mechanisms behind *C. auris* virulence, its ability to colonize various body sites, and its resistance to antifungal treatments. These factors collectively contribute to the pathogen’s potential for causing persistent, hard-to-treat infections, particularly in immunocompromised patients. Thus, evaluating the levels of phospholipase and esterase activity in clinical isolates of *C. auris* is essential for elucidating its pathogenic profile and improving therapeutic strategies [18,19,20].

This ascomycete yeast is a haploid species with high genetic variability among clinical isolates from different geographic populations (clades) and limited diversity within each clade. Low intra- and high inter-group variability proves the independent emergence and evolution of *C. auris* in different regions of the world. Initially, four major clades were identified. In 2018, the existence of a potential fifth clade was reported in Iran, and in 2023, a new (sixth) clade of *C. auris* was found in Singapore and Bangladesh. Notably, isolates from Iran were most closely related to East Asian clade II, while isolates from Singapore and Bangladesh were closely related to representatives of the South American clade IV [21,22,23,24]. The above information, along with the species ability to mate and undergo meiosis, as well as the detection of chromosomal rearrangements between different clades [8,25,26], demonstrates the heterogeneity of the global *C. auris* population, the possibility of recombination of genetic material, and the potential for further evolution, increased virulence, and inheritance of acquired molecular mechanisms of drug resistance [8,9]. Thus, the activity lytic enzyme levels, as well as the sensitivity to antifungal drugs of *C. auris,* currently clearly varies between isolates depending on their geographical origin [27].

In 2017, the first known Russian case of candidemia due to *C. auris* was reported [28]. Genome-wide data analysis showed that this strain clustered within South Asian *C. auris* clade I and seemingly represented an independent event of dissemination from the original species range. From 2017 to May 2020, prior to the COVID-19 pandemic, 30 strains of *C. auris* were identified in Russia. During the COVID-19 pandemic, more than 350 clinical isolates of *C. auris* were deposited in the Russian Collection of Pathogenic Fungi (RCPF) (Kashkin Research Institute of Medical Mycology), isolated from patients in intensive care units of various hospitals and the environment of healthcare institutions. At the same time, the population genetic structure of *C. auris* strains circulating in Russia remains practically unstudied to date.

The molecular intraspecies (within-clade) typing of *C. auris* clinical isolates is important for studying their population structure, epidemiology, and transmission dynamics, in order to identify the epidemic genotype and the origin of *C. auris* outbreaks. Currently, three approaches are most commonly used for molecular intraspecies typing: whole-genome sequencing (WGS), amplified fragment length polymorphism (AFLP), and microsatellite (or short tandem repeat (STR)) length polymorphism analysis. However, WGS is expensive and labor-intensive, and AFLP is a complex technique with results obtained by different research groups being difficult to interpret and compare, while STR-based genotyping has proven to be an effective method for assessing population variation, comparable in efficiency to WGS [29,30]. The STR assay will allow many laboratories to type *C. auris* during outbreaks, and it may also be useful for the initial classification of large sets of isolates at a subspecies or subclade level. A comparative study of five methods (microsatellite typing, ITS sequencing, AFLP, fingerprinting, and MALDI-TOF MS) showed that microsatellite typing is the tool of choice for investigating *C. auris* outbreaks [31]. For example, in India, comparing microsatellite typing to whole-genome sequencing allowed researchers to trace sources and patterns of hospital-acquired infections, enhancing their understanding of outbreak dynamics [32]. Similarly, in Brazil, microsatellite analysis provided insight into the spread of *C. auris* during a COVID-19 outbreak [33]. Moreover, when the first case of *C. auris* emerged in Taiwan, STR typing allowed researchers to determine the isolate’s genetic clade and clarify its epidemiological origin [34]. Despite these studies, there is a lack of research using microsatellite analysis to investigate *C. auris* in Russia, and this study aims to fill this gap by providing new insights into the population structure and epidemiology of this pathogen in Russian healthcare facilities.

The goal of this study is to examine the genetic relationships, virulence levels, and drug-resistance profiles among *C. auris* isolates from different healthcare facilities, with the aim of determining population diversity and the epidemiological landscape of *C. auris* candidiasis in Russia.

## 2. Materials and Methods

### 2.1. Patients and Specimen Collection

In total, 112 *Candida auris* isolates were included in this study, primarily from cases of fungemia and other invasive mycoses, collected between May 2017 and March 2023 at 18 hospitals in Saint Petersburg, the Leningrad Region, and Moscow. The exception was one isolate obtained from an environmental surface (toilet rim) of the healthcare facility. All *C. auris* isolates were collected by standard methods in the laboratories of the 18 hospitals included in the study and were submitted to Mycological Reference Center based at the Kashkin Research Institute of Medical Mycology, North-Western State Medical University, named after I. I. Mechnikov (Saint Petersburg, Russia), for re-testing and determining sensitivity to antifungal drugs. A total of 111 isolates of *C. auris* were recovered from 106 adult immunosuppressed patients (mean age: 63 years; range: 37–97) both with (*n* = 69, 66%) and without (*n* = 35, 34%) COVID-19 pneumonia. Among these patients, 49% (*n* = 51) were female and 51% (*n* = 53) were male. Patients from neonatal and pediatric intensive care units were excluded from the study. Samples from *C. auris* cases were obtained from various specimen sources, including blood, urine, and other sites (see Table 1 for an overview of isolates). For five patients, two consecutive *C. auris* strains, isolated from different localizations (urine, blood) at intervals of 1–2 months, were included in the study. Additionally, to assess result reproducibility, two consecutive *C. auris* strains, isolated from the same site over a short observation period, were included from two patients. In the Section 3 (Figure 3), for the reader’s convenience, samples from the same patient are highlighted in the same color.

### 2.2. Preliminary Identification and Growth Media

The yeast strains of *C. auris* used in this study were deposited in the Russian Collection of Pathogenic Fungi (RCPF) and maintained through traditional subcultivation methods (periodic reseeding on fresh agar media) until use. The preliminary identification of the yeast was performed using matrix-assisted laser desorption ionization time-of-flight (MALDI-TOF) mass spectrometry with an AutoFlex Speed MALDI-TOF MS (Bruker Daltonics, Bremen, Germany), followed by confirmation through the sequencing of the internal transcribed spacer (ITS) rDNA region as universal DNA barcoding for fungi. For all experiments in this study, 24-h cultures grown on Sabouraud dextrose agar (Research Center for Pharmacotherapy, Russia) supplemented with chloramphenicol and incubated at 37 °C were used.

### 2.3. DNA Extraction and Molecular Identification (Internal Transcribed Spacer Region (ITS) Sequencing)

Total genomic DNA was extracted from *C. auris* isolate cultures using the Monarch^®^ HMW DNA Extraction Kit (New England Biolabs Inc., Ipswich, MA, USA) according to the kit’s instructions. The amplification of the internal transcribed spacer (ITS) region of the ribosomal subunit was carried out using fungal general primers: ITS1 (5′-TCCGTAGGTGAACCTGCGG-3′) and ITS4 (5′-TCCTCCGCTTATTGATATGC-3′) (Sinthol, Russia) [35]. PCR reaction was performed in a final volume of 50 µL containing 5 µL dNTP mix (2.5 mM), 5 µL 10× reaction buffer (750 mM Tris-HCl (pH 8.8), 200 mM (NH_4_)_2_SO_4_, 0.1%Tween 20), 2 µL of each primer (5 pM), 5 µL 25 mM MgCl_2_, 2.5 µL DNA template, 0.2 µL Taq DNA polymerase (5000 U/mL) (New England BioLabs, Ipswich, MA, USA), and 28.3 µL nuclease-free water. A negative control (water instead of fungal DNA template) was included. The amplification program consisted of an initial denaturation at 95 °C for 5 min, followed by 35 cycles of 94 °C for 30 s, 56 °C for 30 s, and 72 °C for 1 min, with a final extension at 72 °C for 10 min. PCR products were analyzed by 6% polyacrylamide gel electrophoresis to evaluate size and quality. After that, PCR products were purified using GeneJET PCR Purification Kit (Thermo Scientific, Foster City, CA, USA) and used as the template for sequencing PCR. The sequencing reaction was carried out using the BigDye Terminator v 3.1 Cycle Sequencing Kit (Applied Biosystems, Foster City, CA, USA) with the aforementioned forward and reverse primers, followed by analysis of the reaction products on an Applied Biosystems 3500 Genetic Analyzer. For species identification, GenBank Basic Local Alignment Search Tool (BLAST) search (http://www.ncbi.nlm.nih.gov/BLAST/Blast.cgi, accessed on 20 November 2023) was used. Pairwise sequence alignment was performed using the Seaview program version 4.7 and used to assess the nucleotide polymorphism of the ITS region of all *C. auris* isolates included in the study.

### 2.4. Phospholipase Activity

Sabouraud dextrose agar (Research Center for Pharmacotherapy, Russia, supplemented) with 0.005 mol/L CaCl_2_, 1 mol/L NaCl, and 8% sterile egg yolk emulsion (Condalab, Madrid, Spain) was used to assess the phospholipase activity of *C. auris* isolates. Yeast cell inoculates at a concentration of 10^6^ CFU/mL (equivalent to McFarland 0.5 standard) were prepared from 24-hour-old cultures, and 10 µL of these inoculates were spotted onto the surface of the agar test medium. The plates were incubated at 37 °C for 3 days. *C. albicans* (ATCC 14053) was used as the positive control. Phospholipase activity (Pz) was measured as described by Price et al. [36]. According to this definition, the Pz value was expressed as the ratio of the colony diameter to precipitation zone plus the colony diameter. It was scored as follows: no phospholipase activity (1), weak phospholipase activity (0.9–0.99), medium phospholipase activity (0.8–0.89), strong phospholipase activity (0.7–0.79), and high productivity (<0.69). The assay was performed in triplicate for each isolate.

### 2.5. Esterase Activity

Agar medium for assessing esterase activity was prepared using 10.0 g Bacteriological Peptone (Condalab, Spain), 5.0 g of NaCl, 0.1 g of CaCl_2_, 15.0 g of European Bacteriological Agar (Condalab, Spain), and 1000 mL of distilled water. After autoclaving, the medium was cooled to approximately 50 °C, and 5 mL of sterile Tween 80 (Sigma-Aldrich, St. Louis, MO, USA) was added. The final pH of this medium was 6.8. Yeast cell inoculum was prepared as described above. The seeded agar plates were incubated at 35 °C and monitored daily for 10 days. Esterase activity was assessed similarly to phospholipase activity.

### 2.6. Antifungal Susceptibility Testing

Antifungal susceptibility testing was performed using the Sensititre™ YeastOne™ YO9 AST Plate (Thermo Fisher Scientific, USA) according to the manufacturer’s instructions. The susceptibility testing panel consists of the following 9 antifungal drugs: micafungin, caspofungin, 5-flucytosine, posaconazole, voriconazole, itraconazole, fluconazole, anidulafungin, and amphotericin B. Stock inoculum suspensions of the yeast were obtained in 5 mL of sterile 0.145 mol/L saline (8.5 g/L NaCl; 0.85% saline) from five colonies of ~1 mm in diameter of 24-h *C. auris* cultures on Sabouraud dextrose agar at 35 °C. The turbidity of each yeast suspension was adjusted by the spectrophotometric method as described in the M27-A4 guidelines [37] initially to a stock suspension concentration of 0.5 McFarland standard (1 × 10^6^ cells per mL) followed by the dilution of the stock suspension to 5.0 × 10^2^ to 2.5 × 10^3^ cells per mL. All experiments included the quality control strain *Candida parapsilosis* (ATCC 22019). Minimum inhibitory concentrations (MICs) were determined colorimetrically after plate incubation at 35 °C for 24 h. Since the Clinical and Laboratory Standards Institute (CLSI) does not provide specific antifungal breakpoints for *C. auris*, categorical results were interpreted according to the tentative MIC breakpoints for *C. auris* published by the Centers for Disease Control and Prevention (CDC): ≥32 μg/mL for fluconazole; not available for voriconazole and other second-generation triazoles (posaconazole and itraconazole); ≥2 μg/mL for amphotericin B and caspofungin; ≥4 μg/mL for micafungin and anidulafungin; and not available for 5-flucytosine (https://www.cdc.gov/fungal/candida-auris/recommendations.html, accessed on 20 November 2023).

### 2.7. Microsatellite Length Polymorphism Genotyping (Short Tandem Repeat Analysis)

Microsatellite analysis of the Russian *C. auris* population by STR typing was performed as previously described, with modifications [29]. A set of primers, M2a, M2b, M2c, M3-1a, M3-1b, M3-1c, M3-2a, M3-2b, M3-2c, M9a, M9b, and M9c (Sinthol, Russia), proposed by de Groot et al. [29] was employed to target 12 STR loci with repeat sizes of two, three, or nine nucleotides (Table 2). *C. auris* DNA was extracted as described above. Polyacrylamide gel electrophoresis was performed to check the quality of the isolated DNA and subsequent PCR products.

The PCR mixture, assembled on ice, contained 75 mM Tris-HCl (pH 8.8), 20 mM (NH4)_2_SO_4_, 0.01% Tween 20, 0.25 mM of each dNTP, 2.5 mM MgCl_2_, 1 U of Taq polymerase (New England BioLabs, Ipswich, MA, USA), 1.20 pM of each forward and reverse primer, 25 ng of DNA template, and water to a total volume of 25 µL. A separate PCR reaction was performed with each pair of primers on the following thermal protocol: 5 min of denaturation at 95 °C, followed by 35 cycles of 95 °C for 30 s, 60 °C for 30 s, and 72 °C for 1 min, and a final extension at 72 °C for 10 min. For the STR analysis, PCR products were purified using the GeneJET PCR Purification Kit (Thermo Scientific, Foster City, CA, USA) according to the instructions. After that, purified samples were diluted 100 times with water, and 1 µL of the diluent together with 8.8 µL of formamide and 0.2 µL of GeneScan™ 600 LIZ™ DNA Size Standard v2.0 (Thermo Scientific, Foster City, CA, USA) were denatured for 1 min at 96 °C. Capillary electrophoresis was performed using a standard fragment analysis program on an Applied Biosystems 3500 Genetic Analyzer (Applied Biosystem, Waltham, MA, USA).

### 2.8. Data Analysis

ITS rDNA sequence alignment was performed using the SeaView software (version 4.7). A phylogenetic tree of the ITS sequences was constructed with the maximum likelihood algorithm (PhyML). The copy number of the twelve STR markers in the *C. auris* isolates was determined using GeneMapper Software 5 (Applied Biosystems), and the allele sizes were rounded up. Relatedness among isolates was analyzed with the MEGA 11 software, employing the unweighted pair group method with arithmetic mean (UPGMA) using a distance matrix. The genetic relationships between strain genotypes were visualized through Principal coordinate analysis (PCoA). This analysis was conducted in R 4.1.2, using the genetic distance metric of Bruvo et al. [38] via the POLYSAT v. 1.2-1 package [39].

Statistical analyses were performed in SPSS software version 20.0 (SPSS Inc., Chicago, IL, USA). Differences in phospholipase activity between isolates from different specimen sources were evaluated using the non-parametric Mann–Whitney U test. Spearman’s correlation coefficient was employed to assess the correlation between enzyme activity and antifungal MICs in *C. auris* isolates. A *p*-value of < 0.05 was considered statistically significant (the threshold for detecting differences). In addition, MIC_50_ (the MIC value inhibiting half of the isolates) and MIC_90_ (the MIC value inhibiting 90% of the isolates) were calculated using MIC values rounded up to the next highest concentration.

## 3. Results

### 3.1. Sequence Analysis of ITS Genes

**Figure 1:**
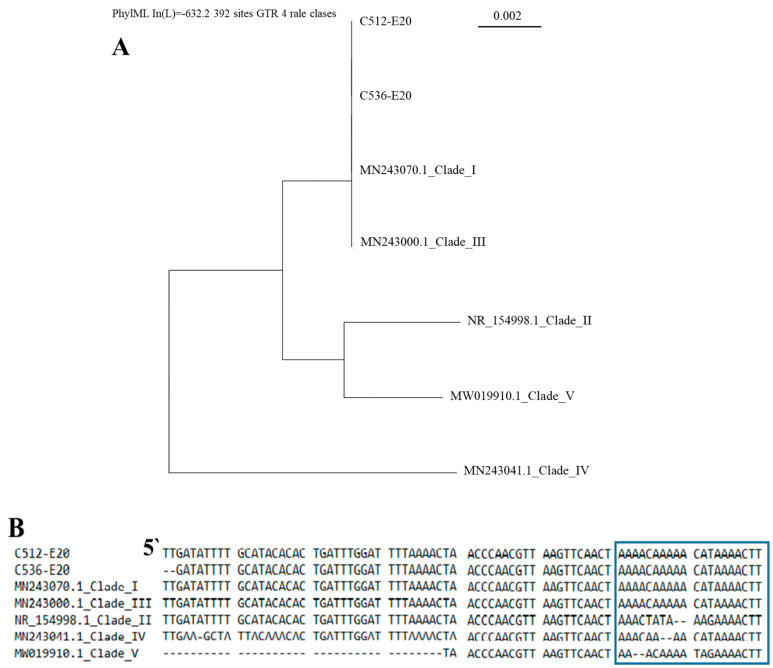
The nucleotide polymorphism of the ITS rDNA region of different *C. auris* clades. (**A**) A phylogenetic tree generated by the maximum likelihood analysis of *C. auris* ITS rDNA sequences. (**B**) ITS rDNA sequence alignment. A clade-specific area of the ITS region is highlighted in a frame. Five typed strains from five different clades [30], submitted with their GenBank accessions, and two strain studied in this study, represented by the numbers C512−E20 and C536−E20. Four ITS-based clusters are shown: clades I and III, clade II, clade IV, and clade V.

The analysis of ITS rDNA sequences using BLAST confirmed the species identification of all study isolates as *C. auris* with 99–100% identity. It has been shown that the nucleotide polymorphism of the ITS region of *C. auris* isolates has clade-specific features [31,40], allowing the differentiation of three out of five known clades. The exceptions are *C. auris* clades I and III, which possess identical ITS regions. Typing of *C. auris* clades has clinical value, as it can help to assess virulence, transmission, and drug resistance potential of a strain [8,21]. *C. auris* isolates from patients in Russia had an identical ITS sequence and belonged to the same ITS-based cluster, which included clades I and III (Figure 1). Previously, based on the WGS sequence, we determined that strain C1-E20, a causative agent of the first known clinical case of *C. auris* candidemia in Russia, belongs to clade I [28]. These data suggest that all *C. auris* isolates that are STR-typed in this study are of South Asian origin.

### 3.2. Phospholipase and Esterase Activity

**Figure 2:**
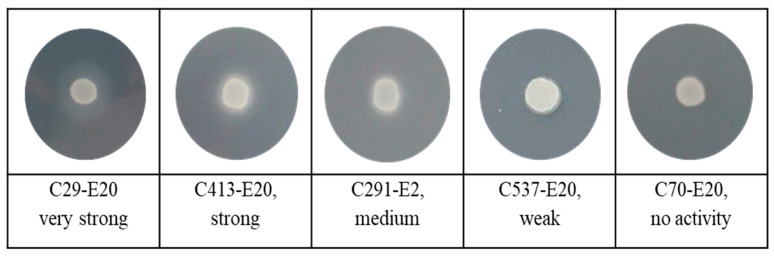
Examples of different groups of phospholipase activity of *Candida auris* strains.

**Figure 3:**
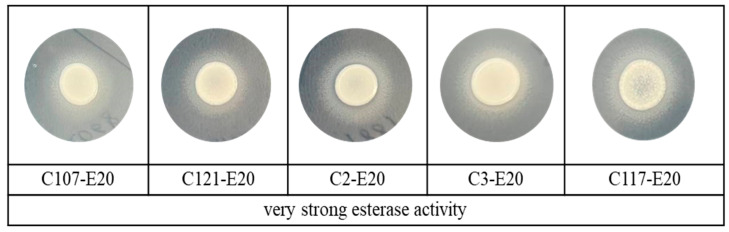
Esterase activity isolates of *Candida auris*.

**Figure 4:**
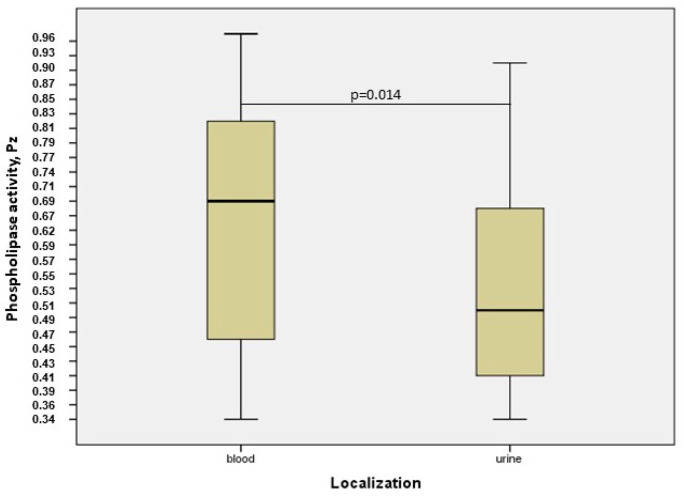
Phospholipase production by *C. auris* strains isolated from blood and urine.

**Figure 5:**
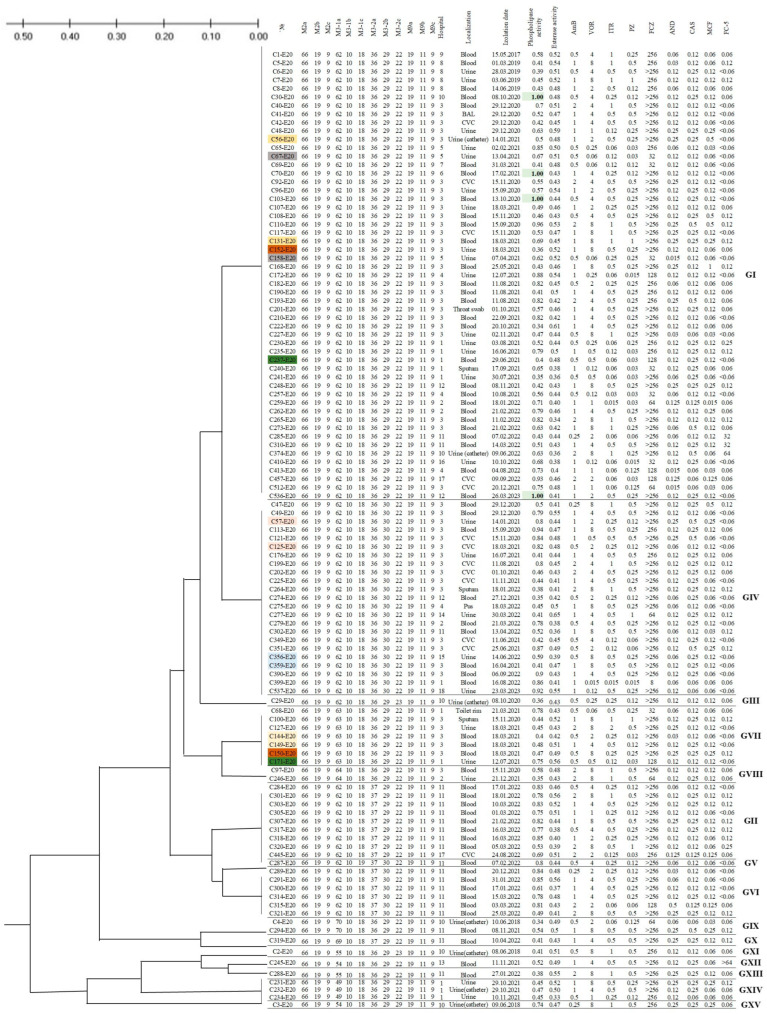
A phylogenetic tree of 112 *Candida auris* strains isolated in Russia, generated using the STR Bruvo distance matrix with the MEGA 11 software. The UPGMA dendrogram illustrates the genotyping of the 112 *Candida auris* isolates based on the STR data, with supplementary information on antifungal susceptibility profiles and enzymatic activity. The numbers of strains isolated from biomaterial of the same patients are highlighted in the same color. The absence of phospholipase activity is indicated by pink color.

Figure 2 and Figure 3 illustrate the phospholipase and esterase activities of *C. auris* produced on selected solid mediaThe Russian clinical *C. auris* isolates exhibited varying abilities to produce phospholipase. Although phospholipase activity was predominantly strong and very strong (74.1%), 8.03% of the strains showed low or no activity (Table 3). At the same time, esterase activity among all strains of *C. auris* was very strong with a precipitation zone from 0.32 to 0.62 when measured after 10 days. For the comparative analysis of enzymatic activity, *C. auris* strains were grouped as follows: isolates from urine and urinary catheters formed one group, isolates from blood and central venous catheters formed another group, and isolates from other loci were combined into the “other localization” group. Data analysis by the Mann–Whitney U test showed a significant difference in extracellular phospholipase activity between *C. auris* strains isolated from blood and those isolates from urine (*p* = 0.014), with a higher fermentative activity in urine strains, as shown in Figure 4. Detailed information on enzymatic activity profiles of all studied *C. auris* isolates is presented in Figure 5.

### 3.3. Antifungal Susceptibility Testing Profiles (Patterns)

Table 4 shows the results of MIC ranges, MIC_50_ and MIC_90_ of antifungal drugs against 112 *C. auris* isolates. Almost all *C. auris* isolates in the present study were resistant to fluconazole (with the exception of one strain). Moreover, 93% of the studied strains had an MIC of ≥256 μg/mL. For second-generation triazoles, there are currently no established MIC breakpoints for any species of the genus *Candida*. Voriconazole (for which specific antifungal breakpoints have not been established) had MIC_50_ and MIC_90_ of 4 and 8, respectively. Among the fluconazole-resistant *C. auris* isolates, 17% (*n* = 19) showed cross-resistance to amphotericin B (MIC ≥ 2 μg/mL), and 3.6% (*n* = 4) exhibited high MIC values for 5-flucytosine (≥32 μg/mL). Notably, among all the samples, only one isolate was resistant to the three classes of antifungal drugs (azoles, polyenes, pyrimidines) (0.89%). Echinocandins such as anidulafungin, micafungin and caspofungin showed strong activity (MIC ≤ 1 mg/mL) against 100% of *C. auris* strains. All isolates had wild-type echinocandin MICs. The antifungal susceptibility pattern with the STR genotype of 112 *C. auris* clinical isolates is presented on Figure 5. Additionally, correlation analysis results using Spearman’s coefficient revealed no statistically significant association between virulence factor (phospholipase and esterase) and antifungal susceptibility profiles (*p* > 0.05); see Table 5.

### 3.4. Microsatellite Length Polymorphism Genotyping

A total of fifteen distinct STR types were identified among 112 *C. auris* strains from 18 different hospitals in Russia (Table 6, Figure 5), differing from each other in one or two markers. A total of 53 studied strains of *C. auris* from 14 healthcare facilities in Saint Petersburg, the Leningrad Region, and Moscow, isolated from 2017 to 2023, were classified as the STR genotype I. These STR genotype I strains also included the first known isolated strain of *C. auris* (C1-E20) in Russia. The second most prevalent genotype was STR type IV, which differs from STR type I by a single repeat in the M3-IIb marker, suggesting it may represent a microevolutionary variant within the population. STR type IV was represented by 23 isolates from nine hospitals in three regions. Several STR genotypes (such as II, VI, VII, and XIV) were represented predominantly (or entirely) by single-hospital strains isolated during the COVID-19 pandemic. These data suggest a nosocomial origin within the STR type of *C. auris* isolates and their subsequent circulation within the healthcare settings. The remaining genotypes were represented by single strains from various hospitals. The most variable of the analyzed markers was M3-Ia, which exhibited eight distinct variants within the Russian population. In contrast, eight microsatellite markers (M2a, M2b, M2c, M3-Ib, M3-Ic, M9a, M9b, and M9c) showed no variation in STR number among all *C. auris* isolates included in this study.

**Figure 6:**
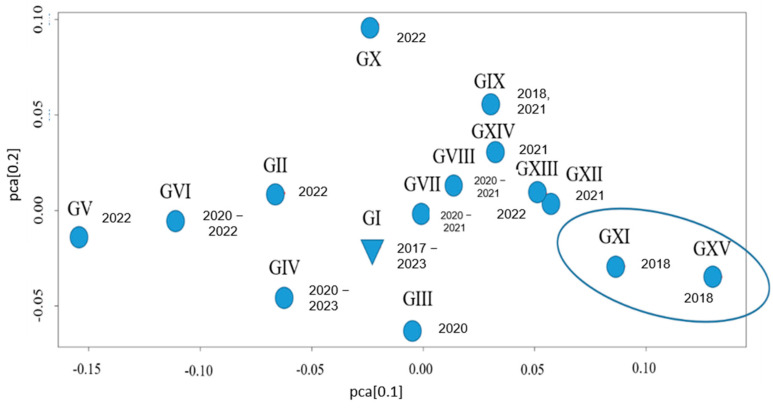
PCoA analysis using Bruvo et al. distance of genetic diversity between *C. auris* isolates included in this study. G-STR genotype; next to the genotype are the years in which the strains were isolated. The genotypes highlighted in the oval have the greatest distances from the most represented genotype in the population.

Principal coordinate analysis (PCoA) of the Bruvo et al. distances between STR genotypes of the *Candida auris* strains from the Russian population revealed low variability among the studied isolates (Figure 6). The plot of the first two principal coordinates represents a “cloud” without the distinct clustering of strains, though small outliers corresponding to individual genotypes are present. The first identified strain of *C. auris* in Russia (C1-E20), classified as STR genotype I, is positioned near the center of the “cloud,” reflecting its foundational role within the studied population. Clustering of genotypes based on the year of strain isolation suggests the potential vectors of intrapopulation microevolution of *C. auris* strains in Russia.

When analyzing consecutive *C. auris* strains isolated from the different loci of the same patient over a long observation period, three pairs of isolates (C56-E20 and C131-E20, C57-E20 and C125-E20, C356-E20 and C359-E20) showed identical STR genotypes. However, in two patients (C150-E20 and C152-E20, C171-E20 and C237-E20), the genotypes of clinical isolates obtained from different locations were different. This may be attributed to either the microevolution of the strain during persistence in the host or possible reinfection through nosocomial transmission. The adequacy and reproducibility of the methods used were confirmed. For instance, independent consecutive isolated strains (C67-E20 and C158-E20, C144-E20 and C149-E20) from the same patient’s biomaterial after a short time interval had an identical STR genotype and antifungal susceptibility and enzymatic activity patterns.

In a comparative analysis of STR genotypes with the date of isolation of the strains, the source, susceptibility drug profile, and phospholipase and esterase activity, no significant correlations were identified. However, the phenotypic absence of phospholipase activity was observed exclusively among strains belonging to the STR genotype I.

## 4. Discussion

*C. auris* is an emerging yeast pathogen; currently, it is one of the four fungi classified as a high-priority health threat (according to WHO).

This study is the first to describe the population structure (based on microsatellite marker analysis), antifungal susceptibility patterns, and proteolytic enzyme expression of *C. auris* strains isolated in Russia. The genetic structure of the population was determined using the STR typing approach for *C. auris*, proposed by de Groot T. et al. [29,41]. This genotyping technique has proven to be a simple and high-resolution screening tool for leassessing genetic diversity in *C. auris*, with results comparable to whole-genome sequencing (WGS) analysis, as variations in STR markers correlate with single-nucleotide polymorphism (SNP) differences [30,32]. The discriminatory power of the proposed STR analysis enables the identification of both inter-clade genetic differences and relationships between isolates within a clade, such as those observed in outbreak clusters. The authors of the typing technique suggest that small inter-strain variations (copy number < 5) in STR markers with a repeat unit of 2–3 nucleotides should be considered as microevolutionary changes within an outbreak. However, more copy number variations in these STR markers or variation in a M9 STR marker strongly indicates that the isolates are not related [29].

During the analysis of 12 STR markers, all studied isolates were grouped into 15 genotypes, similar to the *C. auris* genotypes of South Asian origin (clade I), identified in a number of studies by our colleagues [29,33,42]. Thus, based on the ITS polymorphism assessment, STR typing, and previous WGS analysis of the C1-E20 strain [28], we conclude that all Russian clinical isolates of *C. auris* belong to clade I and share a common South Asian ancestor.

The *C. auris* strain (C1-E20) was assigned to the most prevalent I STR genotype. The long period of I STR genotype strain isolation and their presence in the hospitals in all studied regions of the country allow us to consider it as the first genotype introduced into the territory of Russia. Genetic distances estimated based on the PCoA analysis (Figure 6) and small variations (copy number < 5) in STR markers between I and VIII genotypes indicate the intrapopulation microevolution of strains derived from genotype I in different hospitals. In addition, this genotype in Russia is apparently associated with the formation of nosocomial outbreaks of invasive candidiasis that are difficult to eradicate. The independent introduction of other strains (IX–XV genotypes), highly divergent from the I STR genotype, cannot currently be denied. A small number of isolates (*n* = 1–3) belonging to these genotypes suggests a community-acquired origin, outside of the healthcare system, and their lack of predisposition to nosocomial spread (the development of infectious processes with reduced contagiousness). However, to form conclusions, the further monitoring of clinical isolates of *C. auris* using this analysis scheme is necessary.

Studies by other authors also demonstrate the intrapopulation dominance of one specific STR genotype. Thus, when studying the molecular epidemiology of an outbreak of invasive candidiasis (*n* = 71), which lasted for 1.5 years in one of the medical institutions of Kuwait, and comparing STR typing data of clinical isolates of *C. auris* from other hospitals in the country, it was shown that all studied *C. auris* isolates belong to South Asian clade I, are very closely related to isolates from India and Oman, and differ only at one M3-Ia locus [43]. In total, three STR genotypes variable for M3-Ia were isolated in Kuwait, with the dominance of one genotype 1c, associated with the first isolate of *C. auris* documented in Kuwait, and the remaining two genotypes were represented in strains from single patients. The researchers concluded that clade I isolates of *C. auris* have been transmitted over many years in Kuwait, undergoing microevolution in various healthcare facilities. This process led to the emergence of three distinct genotypes of clade I, with genotype 1c being specifically linked to nosocomial outbreaks of invasive candidiasis.

It should be noted that the M3-Ia locus is the most variable marker, both according to the results of our study and global research, reflecting microevolutionary changes in outbreak studies [29,42,44]. Our study showed the simultaneous presence in the same patient of *C. auris* strains with distinct STR genotypes variable at the M3-Ia locus (per one repeat), which were isolated from different biological materials (blood, urine, etc.). A similar case was previously described in a study by our colleagues that studied the nosocomial transmission dynamics of *C. auris* in a referral chest hospital in India. In patient D, *C. auris* strains were found from different body sites (ear, nose, groin), which differed only in copy number (2 or 4) of the M3-Ia locus [32]. However, despite its variability and mutational plasticity, it can possibly serve as a marker of adaptive evolution and influence the transmissibility, epidemic potential, and disease emergence of *C. auris* isolates.

The virulence of the polyphyletic genus *Candida*, to which *C. auris* belongs, is also associated with the production of multiple hydrolytic enzymes that facilitate invasion into host tissues by damaging cell surface structures and degrading various host proteins [19,45]. Several studies have demonstrated that the higher expression of phospholipase and esterase correlates with the higher virulence of strains, confirming their roles as key pathogenicity factors. *C. auris* strains tested across different geographical regions exhibit-varying levels of phospholipase and esterase activities. This fact may be associated with both the geographical clade of the species and the strain-specific characteristics [46]. Thus, of 107 isolates from two cities in Colombia, phospholipase activity was observed in 67.3% of 107 *C. auris* isolates [47]. Various studies on the phospholipase activity of *Candida* species demonstrate a possible correlation between the locus of isolates and enzyme activity [20,48]. In our study, *C. auris* strains isolated from urine have a higher phospholipase activity than that of strains from blood. However, esterase activity was uniformly high across all strains.

Currently, the recommended first-line drugs for treating invasive candidiasis in the intensive care units (ICUs) are limited to echinocandins and azoles. Taking into account that the resistance of *C auris* isolates to fluconazole is nearly universal, especially among clade I [49], de-escalation therapy for invasive candidiasis caused by this pathogen is limited to one class of antifungal drugs [50]. However, the frequency of resistance to echinocandins in clinical *C auris* isolates is increasing worldwide. According to published data, it is reported that echinocandin resistance has increased from 0% to 4% in New York from 2016 to 2020 [51]. The current results showed that the vast majority of Russian *C. auris* strains are resistant to fluconazole, while resistance to echinocandins was not detected among them. Therefore, at present (based on the high fungicidal activity against most strains of *C auris*), echinocandins are the only alternative for effective and safe therapy of invasive candidiasis in ICU conditions.

Amphotericin B and 5-flucytosine are less recommended due to potential toxicity as well as poor activity against urinary tract infections for amphotericin B and rapid development of resistance in monotherapy for 5-flucytosine. However, the narrow spectrum of antifungal drugs that are potentially effective against infections caused by *C. auris* does not preclude their use in clinical practice [50]. In most populations, resistance to amphotericin B in *C. auris* isolates ranges from 0% to 30% [52,53]. The resistance of Russian *C. auris* strains falls within this range and is 17%. There is no CDC advisory for flucytosine breakpoints, and we note four *Candida auris* isolates with an MIC value of ≥32 μg/mL. Flucytosine is used less frequently compared to other antifungal agents; fewer studies have been conducted to detect *C. auris* resistance. These four strains were isolated from patients from intensive care units of three hospitals in Moscow. Two strains, C285-E20 and C310-E20, were isolated from different patients placed in different departments of the same hospital with a one-month difference in isolation. Two other strains, C374-E20 and C245-E20, were isolated from non-communicating hospitals. Unfortunately, we have no data on the possible flucytosine therapy administered to these patients.

Although preliminary MIC breakpoints are suggested by the CDC for only five drugs (fluconazole, amphotericin, anidulafungin, caspofungin, and micafungin), it is also important to monitor susceptibility levels for other antifungal drugs in order to determine, in a timely manner, the trends in increasing resistance in this potentially multidrug-resistant pathogen.

The current study has a number of limitations. First, the studied *C. auris* strains had limited metadata such as geographic region, site of infection, healthcare facilities, gender, and age of the patient. There are no data on patient outcome, possible pharmacotherapy (antifungal medications), and epidemiological anamnesis. Second, this research was retrospective; therefore, the environmental studies of healthcare facilities were not carried out according to the recommendations. This fact makes it difficult to determine the sources and routes of pathogen transmission within hospitals, as well as predisposing factors for the nosocomial persistence of strains and the emergence of genetic diversity (microevolutionary changes). Third, because there are no universally accepted breakpoints for all antifungals against *C. auris*, antifungal susceptibility for some drugs has been interpreted based on the MIC and by comparison with breakpoints for other species. Fourth, the study is limited to three geographical regions; since at the time when this study was conducted, there was no information on *C. auris* isolation in hospitals of other cities.

## 5. Conclusions

This study is the first to explore the genetic diversity of *Candida auris* strains circulating in Russian healthcare facilities, based on short tandem repeat (STR) lengths, antifungal susceptibility profiles, and enzymatic activity. The utility of STR analysis for epidemiological monitoring, tracking global emergence, and understanding transmission dynamics of this pathogen is demonstrated. Our findings reveal the predominance of a single STR genotype (I) in most hospitals across Russia and identify a specific genotype (M3-Ia locus) that is presumably linked to transmissibility, epidemic potential, and the occurrence of nosocomial outbreaks of invasive candidiasis caused by *C. auris*. Ultimately, the integration of genetic information into anti-epidemic measures, along with the appropriate selection of antifungal agents based on susceptibility profiles, will aid in controlling the spread and transmission of this dangerous yeast pathogen in Russia.

## Figures and Tables

**Table 1:** The major characteristics of *Candida auris* strains.

Characteristic	No. (%)
**Localization**	
Blood	60 (53.57%)
Central venous catheter	13 (11.60%)
Urine	25 (22.30%)
Urine catheter	7 (6.25%)
BAL	1 (0.90%)
Sputum	3 (2.68%)
Pus	1 (0.90%)
Throat swab	1 (0.90%)
Environmental object	1 (0.90%)
**Isolation year**	
2017	1 (0.90%)
2018	3 (2.68%)
2019	4 (3.57%)
2020	18 (16.07%)
2021	49 (43.75%)
2022	35 (31.25%)
2023	2 (1.78%)

**Table 2:** Amplification primers used for microsatellite genotyping of *Candida auris* isolates.

Marker	Primer Sequence (5′–3′)		Repeat Unit
	Forward Primer	Reverse Primer	
M2a	FAM-GCAACATCCTGAGCAGTATCAC	GGTGTTGACGTGCCCAAATATGC	AG
M2b	HEX-CCACTCCGTTTTGGGTCTG	AGAGAATCTACAAATGTGTCGC	AG
M2c	ROX-CTGTTTCTGTGGCAGGCTTCC	GCCACGTTTCACYGCYACCAT	AG
M3-Ia	FAM-GCATGGATCAACAGCTAACAG	AGTGCCAGGCTGTGTACTTTTG	CAA
M3-Ib	HEX-CATCCTAACGCTGGCTCTTC	GGYTTTGAGGYTGCCCTAGC	CAA
M3-Ic	ROX-GCAACTACGCATTGTGTATTC	CTAACAGAGGATTTCAATTGCC	TTA
M3-IIa	FAM-GTTCAAAATCGCTGACGGTC	GAGATGATGATGGCACTTGC	CTA
M3-IIb	HEX-GTGAATGGAGCACCACAACCAG	GCGCAAATGACTGGCCCATG	GTA
M3-IIc	ROX-GTGATGAGCGCACTACACAGG	GGCGAAGAAACGGTGAGTAC	CTA
M9a	FAM-CTTGTCTAGTTTGCGATCTACGC	GAGACTGCCAAGCCAAGC	GATGATGAA
M9b	HEX-CTGCTTACTGGAGACTCTTCC	GATGAGGAGGACGAGGACG	TCATCGTCA
M9c	ROX-GTACGAAATGGGGATAATTGGG	ACCAACCGTGCTATTCTC	TCCTTCTTC

**Table 3:** Phospholipase activity of *C. auris* isolates.

Sample Type	Phospholipase Activity, Pz				
	<0.69 (++++)	0.70–0.79 (+++)	0.80–0.89 (++)	0.90–0.99 (+)	1.00 (−)
Urine (*n* = 32)	25 (78.12%)	3 (9.38%)	3 (9.38%)	1 (3.16%)	0 (0%)
Blood (*n* = 73)	37 (50.68%)	11 (15.07%)	17 (23.29%)	4 (5.48%)	4 (5.48%)
Other localization (*n* = 7)	6 (85.71%)	1 (14.29%)	0 (0%)	0 (0%)	0 (0%)
All (*n* = 112)	68 (60.71%)	15 (13.40%)	20 (17.86)	5 (4.46%)	4 (3.57%)

Activity level: −, Pz  =  1 (no phospholipase activity); +, Pz  =  0.90 to 0.99 (weak phospholipase activity); ++, Pz  =  0.80 to 0.89 (medium phospholipase activity); +++, Pz  =  0.70 to 0.79 (strong phospholipase activity); ++++, Pz  =  < 0.69 (very strong phospholipase activity).

**Table 4:** In vitro antifungal susceptibility profile of *C. auris* isolates (*n* = 112).

Antifungal Drugs	MIC Range (μg/mL)	MIC_50_	MIC_90_	MIC (μg/mL)													
		(μg/mL)	(μg/mL)	≥256	128	64	32	8	4	2	1	0.5	0.25	0.125	≤0.06	0.03	0.015
AmB	0.25–2	1	2							19	57	32	4				
VOR	0.015–8	4	8					33	37	18	6	5	4	4	4		1
ITR	0.015–2	0.5	1							1	21	49	17	8	13	1	2
PZ	0.015–1	0.25	0.5								5	31	40	18	4	11	3
FCZ	8–≥256	256	256	93	6	5	7	1									
AND	0.015–0.5	0.12	0.25									1	20	65	19	4	3
CAS	0.06–0.5	0.25	0.25									8	49	47	8		
MCF	0.015–1	0.12	0.25								1	4	10	55	35	6	1
FC-5	≤0.06–≥64	0.06	0.12			2	2						1	25	80		

AmB—amphotericin; VOR—voriconazole; ITR—itraconazole; PZ—posaconazole; FCZ—fluconazole; AND—anidulafungin; CAS—caspofungin; MCF—micafungin; FC-5—flucytosine.

**Table 5:** Correlation coefficients between virulence factors and antifungal MICs in *C. auris* isolates.

Antifungal Drugs	Virulence Factors (*p*-Value)	
	Esterase	Phospholipase
AmB	−0.002 (0.986)	0.070 (0.462)
VOR	−0.001 (0.992)	−0.220 (0.200)
ITR	0.085 (0.375)	−0.162 (0.087)
PZ	0.096 (0.312)	−0.225 (0.017)
FCZ	0.025 (0.795)	−0.033 (0.726)
AND	0.052 (0.584)	0.018 (0.849)
CAS	−0.075 (0.432)	−0.006 (0.949)
MCF	0.092 (0.332)	0.045 (0.635)
FC-5	−0.086 (0.367)	−0.077 (0.419)

**Table 6:** STR genotypes of 112 *C. auris* isolates of Russia.

Genotype	M2a	M2b	M2c	M3-1a	M3-1b	M3-1c	M3-2a	M3-2b	M3-2c	M9a	M9b	M9c	No. of Isolates	No. of Hospitals
GI	66	19	9	62	10	18	36	29	22	19	11	9	53	14
GII	66	19	9	62	10	18	37	29	22	19	11	9	9	2
GIII	66	19	9	62	10	18	36	29	23	19	11	9	1	1
GIV	66	19	9	62	10	18	36	30	22	19	11	9	23	9
GV	66	19	9	62	10	19	37	30	22	19	11	9	1	1
GVI	66	19	9	62	10	18	37	30	22	19	11	9	6	1
GVII	66	19	9	63	10	18	36	29	22	19	11	9	7	2
GVIII	66	19	9	64	10	18	36	29	22	19	11	9	2	2
GIX	66	19	9	70	10	18	36	29	22	19	11	9	2	2
GX	66	19	9	69	10	18	37	29	22	19	11	9	1	1
GXI	66	19	9	55	10	18	36	29	23	19	11	9	1	1
GXII	66	19	9	54	10	18	36	29	22	19	11	9	1	1
GXIII	66	19	9	53	10	18	36	29	22	19	11	9	1	1
GXIV	66	19	9	49	10	18	36	29	22	19	11	9	3	1
GXV	66	19	9	54	10	18	36	29	29	19	11	9	1	1

## Data Availability

The original contributions presented in this study are included in the article. Further inquiries can be directed to the corresponding author.
